# Simple extraction method using a nontraumatic tube for impacted stool in a diverticulum near the ulcer floor after endoscopic submucosal dissection

**DOI:** 10.1055/a-2081-4829

**Published:** 2023-05-26

**Authors:** Yohei Koyama, Masakatsu Fukuzawa, Fumito Yamanishi, Taisuke Matsumoto, Hayato Yamaguchi, Takashi Kawai, Takao Itoi

**Affiliations:** 1 Department of Gastroenterology and Hepatology, Tokyo Medical University Hospital, Tokyo, Japan; 2 Department of Gastroenterological Endoscopy, Tokyo Medical University Hospital, Tokyo, Japan

With the development of specific knives and traction devices, endoscopic submucosal dissection (ESD) of colorectal tumors such as those that extend into diverticula, which are technically difficult, is becoming possible 1 2 3 4 . A colonic diverticulum is a false diverticulum without a muscular layer, and therefore only the serosa remains after submucosal dissection. To prevent delayed perforation, complete closure of the diverticulum is necessary; however, colonic diverticula often exist in multiples close to each other, and closure of a diverticulum containing impacted stool carries a risk of causing diverticulitis.

An 86-year-old man had a laterally spreading tumor (LST) extending into a diverticulum in the ascending colon ( Fig. 1 ). The LST was resected by ESD using the countertraction technique, with successful en bloc resection achieved without any adverse events. We attempted to close the diverticular part of the ulcer floor using a clip to prevent delayed perforation; however, there was a second diverticulum containing impacted stool close to the ulcer floor ( Fig. 2 a ). We tried to extract the stool using hemostatic forceps and the waterjet function of the endoscope, but were unsuccessful. We therefore placed a nontraumatic tube (MD-PW01Y; Olympus, Tokyo, Japan) ( Fig. 3 ) into the diverticulum, and flushed water through the tube. The stool that had been impacted in the diverticulum was then displaced and successfully and easily extracted using only suction ( Video 1 ). The two diverticula were then closed together with clips, and neither delayed perforation or diverticulitis occurred ( Fig. 2 b, c ). The tumor was histologically diagnosed as a well-differentiated adenocarcinoma that was limited to the mucosal layer, and a curative resection was achieved.

**Fig. 1:**
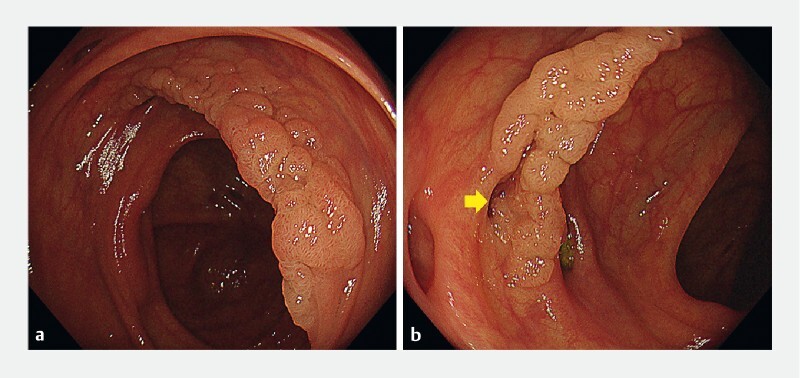
Endoscopic images of the tumor showing: **a** a 40-mm laterally spreading tumor in the ascending colon; **b** multiple diverticula surrounding the tumor, which was extending into a diverticulum (yellow arrow).

**Fig. 2:**
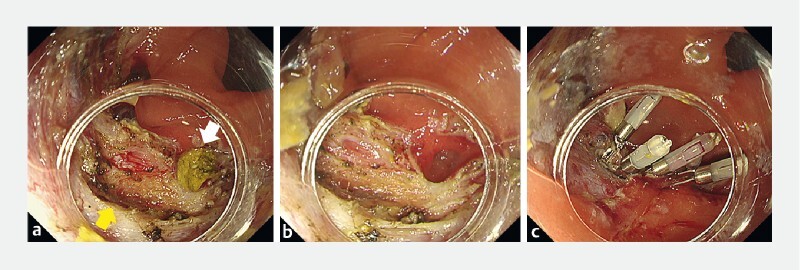
Endoscopic images of the ulcer floor after endoscopic submucosal dissection showing: **a** the diverticular part (yellow arrow) after endoscopic submucosal dissection, and a second diverticulum with impacted stool near the ulcer floor (white arrow); **b** the diverticulum after complete extraction of the impacted stool; **c** the two diverticula after closure together with clips.

**Fig. 3:**
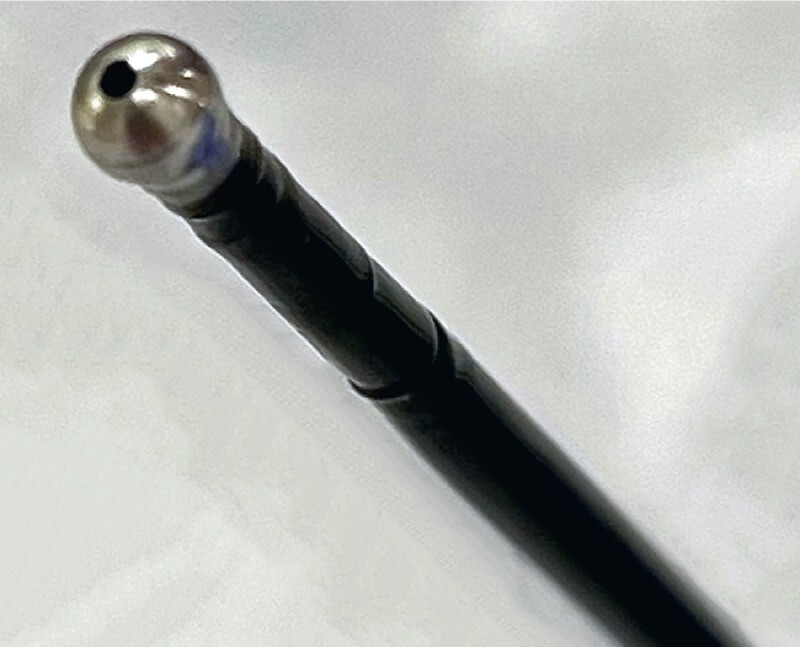
Photograph of the nontraumatic tube, the tip of which is small (2.7 mm) and spherical, and has a spray hole.

**Video 1** A simple extraction method using a nontraumatic tube is used for impacted stool in a diverticulum near the ulcer floor after endoscopic submucosal dissection.


This technique has also been reported to be useful for the removal of hematomas in diverticula during colonic diverticular bleeding 5 . Our present case demonstrates a simple and convenient method for the extraction of impacted stool in a diverticulum.

Endoscopy_UCTN_Code_TTT_1AQ_2AD

## Notes

Endoscopy E-Videos

https://eref.thieme.de/e-videos
*E-Videos* is an open access online section of the journal *Endoscopy* , reporting on interesting cases and new techniques in gastroenterological endoscopy. All papers include a high-quality video and are published with a Creative Commons CC-BY license. Endoscopy E-Videos qualify for HINARI discounts and waivers and eligibility is automatically checked during the submission process. We grant 100% waivers to articles whose corresponding authors are based in Group A countries and 50% waivers to those who are based in Group B countries as classified by Research4Life (see: https://www.research4life.org/access/eligibility/ ). This section has its own submission website at https://mc.manuscriptcentral.com/e-videos
